# NMRtist: an online platform for automated biomolecular NMR spectra analysis

**DOI:** 10.1093/bioinformatics/btad066

**Published:** 2023-02-01

**Authors:** Piotr Klukowski, Roland Riek, Peter Güntert

**Affiliations:** Laboratory of Physical Chemistry, ETH Zurich, 8093 Zurich, Switzerland; Laboratory of Physical Chemistry, ETH Zurich, 8093 Zurich, Switzerland; Laboratory of Physical Chemistry, ETH Zurich, 8093 Zurich, Switzerland; Institute of Biophysical Chemistry, Goethe University Frankfurt, 60438 Frankfurt am Main, Germany; Department of Chemistry, Tokyo Metropolitan University, Hachioji, Tokyo 192-0397, Japan

## Abstract

**Summary:**

We present NMRtist, an online platform that combines deep learning, large-scale optimization and cloud computing to automate protein NMR spectra analysis. Our website provides virtual storage for NMR spectra deposition together with a set of applications designed for automated peak picking, chemical shift assignment and protein structure determination. The system can be used by non-experts and allows protein assignments and structures to be determined within hours after the measurements, strictly without any human intervention.

**Availability and implementation:**

NMRtist is freely available to non-commercial users at https://nmrtist.org.

**Supplementary information:**

Supplementary data are available at *Bioinformatics* online.

## 1 Introduction

Most nuclear magnetic resonance (NMR) studies of proteins remain substantial undertakings that require expert knowledge and weeks or months of manual analysis time. Obviating this complexity can facilitate investigations of protein structure, interactions and dynamics that are currently often deemed to be too demanding. Although there have been many approaches aiming at automating individual steps of NMR spectrum analysis (Würz *et al.*, 2017), a strictly automatic end-to-end method has been developed only recently with our ARTINA algorithm that includes peak picking, assignment and structure determination in completely unsupervised manner (Klukowski *et al.*, 2022). Here, we present NMRtist (https://nmrtist.org), a web platform that offers virtual storage for protein NMR spectra, cloud computing resources and online hosting of applications for the analysis of multidimensional NMR data, in particular deep learning-based solutions implemented in ARTINA. NMRtist is accessed with a web browser and makes it possible to execute complex and computation-intensive spectra analysis methods in a user-friendly manner, such that (i) no expert knowledge (e.g. about high-performance computing or technical details of the algorithms) is required; (ii) no user-side hardware resources are necessary; (iii) on the server side, optimally selected hardware for machine learning and numerical optimization is available that scales to large numbers of users, projects, and application calls; (iv) the output is readily understandable and comprises the graphics and tables typically included in publications on protein NMR structure determinations.

Apart from the immediate practical benefit of automating work-intensive data analysis tasks, NMRtist will allow, due to the acquisition of large-scale datasets from the users, the training of deep learning models for protein NMR spectroscopy at unprecedented scale. To date, most notable manuscripts published in the field refer to <50 spectra in their experimental sections (e.g. in the well-known CASD-NMR competition, only NOESY spectra of 10 proteins were used). In contrast, over 2000 multidimensional NMR spectra were uploaded by the users to the NMRtist servers since the launch of the platform.

## 2 Implementation

**Fig. 1.**
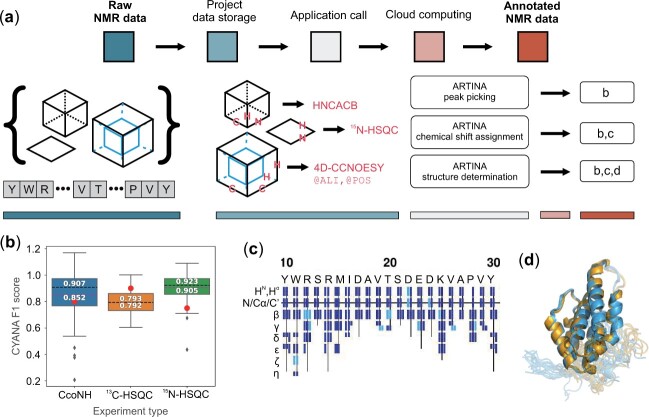
NMRtist data analysis workflow. (**a**) A set of raw 2D/3D/4D NMR spectra and the protein sequence are uploaded to the project data storage. Experiment type and axis labels are specified for each uploaded spectrum. Optionally, additional tags can be provided to facilitate automated data analysis (e.g. @ALI for aliphatic and @POS for positive peaks only). Spectra in project storage are used as input for application calls, yielding outputs (**b–d**). (b) Example figure generated by the ARTINA peak picking application (Section 3). It presents CYANA F1 scores, calculated with the ‘peaks assignscore’ command of CYANA, of automated peak picking of the CcoNH, ^13^C-HSQC and ^15^N-HSQC spectra (red dots) in comparison with those of all spectra of the same type in a benchmark of 1329 spectra (box plot). (c) Example figure generated by the ARTINA chemical shift assignment application. Each column corresponds to a single amino acid with color-coded confidence of automatically assigned shift values (high/low confidence in dark/light shading, respectively). (d) Visualization of protein structures generated by the ARTINA structure determination application. The application outputs two overlaid structure candidates (orange and blue) resulting from two independent CYANA executions (Güntert and Buchner, 2015). Differences between structure candidates indicate either flexible regions or uncertainty in the structure determination

The main logical unit of NMRtist is a *project*. Each project is bound to a single protein sequence and has private storage for experimental data deposition (Fig. 1a). Within the project, the user uploads NMR data [currently, multidimensional NMR spectra in Bruker, XEASY, NMRPipe and UCSF (Sparky) binary formats are supported] and may specify contextual information related to the uploaded data, such as experiment types (currently, 26 types are supported), spectrum axis labels, types of signal folding, etc. Spectra stored in the project may be used as input for application calls (Section 3) that are executed on dedicated CPU/GPU machines (NMRtist computational nodes) in the system. This frees users from the necessity to maintain specialized IT infrastructure yet allowing them to perform advanced computation. The results of application calls are stored in the project storage and made available online to the user.

The architecture of NMRtist has been designed such that the number of handled application calls scales linearly with the amount of hardware resources attached to the system. To assess NMRtist’s throughput capacity, we attached 10 Google Cloud machines to analyze over 1000 3D/4D NMR spectra within 24 h, which is about three orders of magnitude faster than their NMR acquisition time. Due to the scalability and high maximum throughput of the system, the amount of publicly available hardware resources can be adjusted dynamically to meet the demand from the platform users.

## 3 Hosted applications

The NMRtist platform is designed as a hub for disseminating new algorithms and standardized computational routines in computational NMR spectroscopy. Currently, the system contains three applications based on ARTINA models (Klukowski *et al.*, 2022).

*ARTINA peak picking* performs automated cross-peak detection and signal deconvolution using deep residual neural networks (He *et al.*, 2016), and peak list unfolding using kernel density estimation. Its output consists of lists of detected signal positions and intensities in multiple formats, together with quality metrics (Fig. 1b, Supplementary Fig. S1) that assess the uploaded data relative to over 1000 spectra stored in the ARTINA dataset. Additionally, the application performs spectra cross-referencing by detecting systematic shifts in the uploaded dataset (Buchner *et al.*, 2013).

*ARTINA shift assignment* assigns automatically chemical shifts in a set of protein NMR spectra. The application utilizes deep learning for visual spectrum analysis followed by automated assignment with the FLYA algorithm (Schmidt and Güntert, 2012) facilitated by graph neural network chemical shift predictions (Klukowski *et al.*, 2022). The application outputs a list of chemical shifts and their confidence (Fig. 1c), as well as statistics on the use of each spectrum in the assignment process.

*ARTINA structure determination* performs protein structure determination using as input only NMR spectra and the protein sequence. The application performs, if needed, ARTINA peak picking and shift assignment before combined automated NOE assignment and structure calculation with torsion angle dynamics by CYANA (Güntert and Buchner, 2015). Apart from the protein structure in mmCIF and PDB formats, the method yields lists of restraints and chemical shifts in CYANA and NEF (Gutmanas *et al.*, 2015) format, allowing interpretability of its output.

Each ARTINA-based application is available in two modes, *standard* mode corresponding to the original ARTINA implementation (Klukowski *et al.*, 2022) and *test* mode operating with minimal computational resources. The latter increases the usability of NMRtist by enabling short preliminary application calls that may precede the computation-intensive standard ARTINA method execution.

## 4 Conclusions

NMRtist is a versatile online platform that offers major technical advances in automating labor-intensive NMR data analysis, such as peak picking, chemical shift assignment and structure determination. NMRtist encapsulates the complexity of the ARTINA workflow, eliminates any intermediate data and format conversions by the user and employs different types of high-performance hardware as appropriate for each of the subtasks. With NMRtist, NMR data analysis can be completed within hours after the measurements by non-experts, without parameter setting by the user. We believe that the use of NMRtist by the research community will boost the throughput of structural biology research with NMR. In the near future, we plan to extend the set of applications in the NMRtist platform to automated NMR studies of protein–ligand complexes and RNA.

## Supplementary Material

btad066_Supplementary_Data

## Data Availability

The data underlying this article is available at https://nmrtist.org.
