# Supplementary material for: NMRtist: an online platform for automated biomolecular NMR spectra analysis
Source: Bioinformatics. 2023 Feb 1;39(2):btad066. doi: 10.1093/bioinformatics/btad066 (PMC9913044; doi:10.1093/bioinformatics/btad066)
Supplement: btad066_Supplementary_Data [file btad066_supplementary_data.pdf]

A

Spectrum name	Number of peaks				Score (Strong / Weak)				Download [F] - original signal coordinates (folded peaks), [U] - unfolded peak lists		
	Expected	Strong	Weak	All	Precision	Recall	F1	Fm	Strong	Weak	All
C13NOESY	-	8467	9641	10000	-	-	-	-	CSV [F], PEAKS [F], LIST [F] CSV [U], PEAKS [U], LIST [U]	CSV [F], PEAKS [F], LIST [F] CSV [U], PEAKS [U], LIST [U]	CSV [F], PEAKS [F], LIST [F]
CBCANH	520	354	456	801	0.878 / 0.844	0.598 / 0.74	0.711 / 0.788	0.724 / 0.79	CSV [F], PEAKS [F], LIST [F] CSV [U], PEAKS [U], LIST [U]	CSV [F], PEAKS [F], LIST [F] CSV [U], PEAKS [U], LIST [U]	CSV [F], PEAKS [F], LIST [F]
CBCAcoNH	288	283	361	741	0.937 / 0.855	0.921 / 1.071	0.929 / 0.951	0.929 / 0.957	CSV [F], PEAKS [F], LIST [F] CSV [U], PEAKS [U], LIST [U]	CSV [F], PEAKS [F], LIST [F] CSV [U], PEAKS [U], LIST [U]	CSV [F], PEAKS [F], LIST [F]
CcoNH @ALI	451	371	502	1414	0.979 / 0.89	0.805 / 0.991	0.884 / 0.938	0.888 / 0.939	CSV [F], PEAKS [F], LIST [F] CSV [U], PEAKS [U], LIST [U]	CSV [F], PEAKS [F], LIST [F] CSV [U], PEAKS [U], LIST [U]	CSV [F], PEAKS [F], LIST [F]
HCCHTOCSY @ALI	3443	1243	2078	6960	0.778 / 0.708	0.281 / 0.427	0.413 / 0.533	0.467 / 0.55	CSV [F], PEAKS [F], LIST [F] CSV [U], PEAKS [U], LIST [U]	CSV [F], PEAKS [F], LIST [F] CSV [U], PEAKS [U], LIST [U]	CSV [F], PEAKS [F], LIST [F]
N15HSQC	216	158	171	260	1.026 / 1.003	0.751 / 0.794	0.867 / 0.887	0.878 / 0.893	CSV [F], PEAKS [F], LIST [F] CSV [U], PEAKS [U], LIST [U]	CSV [F], PEAKS [F], LIST [F] CSV [U], PEAKS [U], LIST [U]	CSV [F], PEAKS [F], LIST [F]
N15NOESY	-	3131	4323	8396	-	-	-	-	CSV [F], PEAKS [F], LIST [F] CSV [U], PEAKS [U], LIST [U]	CSV [F], PEAKS [F], LIST [F] CSV [U], PEAKS [U], LIST [U]	CSV [F], PEAKS [F], LIST [F]

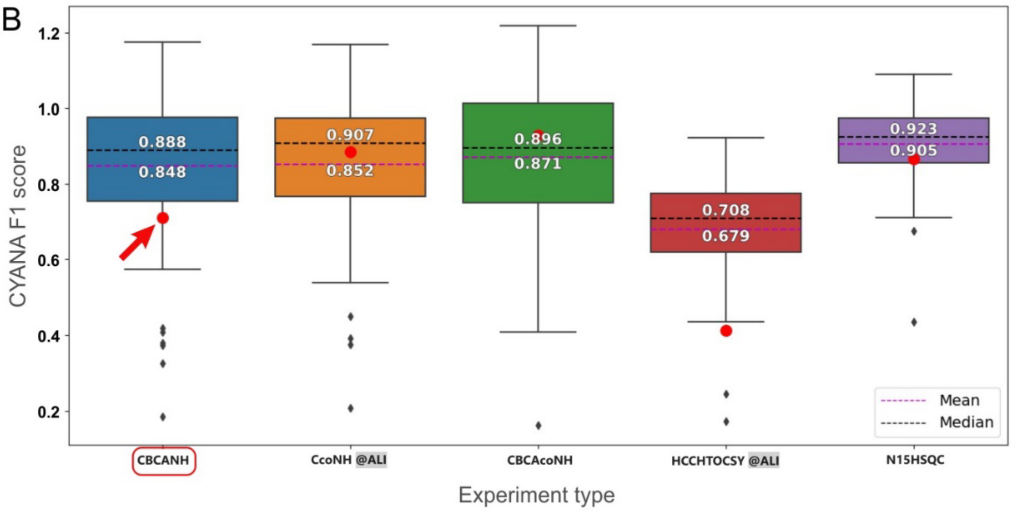

C

Compared peak lists		Relative shift	Score
Reference spectrum	Target spectrum		
CBCANH	N15HSQC	N=0.0, H=0.0	0.72
CcoNH @ALI	N15HSQC	N=0.0, H=0.0	0.8
CBCAcoNH	N15HSQC	N=0.0, H=0.0	1.03
N15HSQC	N15NOESY	N=0.0, HN=0.0	0.28

Figure S1. Example output from ARTINA peak picking application in NMRtist. **A**. Table of automatically detected cross-peaks in each NMR spectrum used as input to the application call. The column 'Expected' presents the theoretical number of peaks that are expected to be present in the spectrum (calculated based on the protein sequence and the experiment type). The subsequent columns, 'Strong', 'Weak', and 'All', inform about the number of peaks detected with high and low confidence, respectively, and the total number of all signals reported by the ResNet model in the spectrum. The middle part of the table (columns 'Precision', 'Recall', 'F1', 'Fm') contains quality metrics calculated for the automatically generated peak lists. The scores are derived from the comparison of each peak list with statistical data extracted from the BMRB database. **B**. Each box plot presents the distribution of CYANA F1 scores calculated for all spectra of the given type in the ARTINA benchmark dataset (1329 spectra in total). The red dot indicates the CYANA F1 score of the user's spectrum analysed by the server. For instance, the CBCANH spectrum (red box) uploaded by the user (CYANA F1 = 0.711) belongs to lowest 25% of all CBCANH spectra stored in the ARTINA benchmark (red dot below the lower quartile). **C**. Result of the automated reference check. If an uploaded spectrum shares two dimensions with ^{13}C-HSQC or ^{15}N-HSQC experiments, the method verifies automatically the possible presence of systematics shifts in the data. For example, the user's CBCANH (red box) shares the H and N dimensions with the ^{15}N-HSQC experiment (violet box). By comparing the output of the automated peak picking of both spectra, NMRtist didn't detect any systematic reference shifts (N=0.0, H=0.0), and this result is provided with the confidence score 0.72.

A

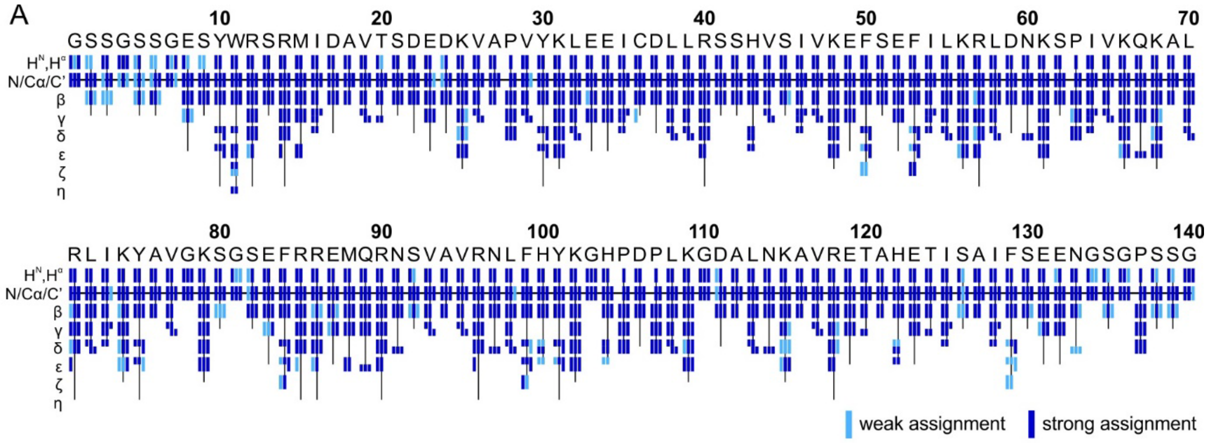

B

Spectrum name	Expected	Assigned expected	Measured	Assigned measured	Ratio	Assigned peak lists
C13NOESY	5908	3553 (60.14%)	9968	2848 (28.57%)	1.2	XEASY Sparky
CBCANH	519	401 (77.26%)	497	358 (72.03%)	1.1	XEASY Sparky
CBCAcoNH	260	241 (92.69%)	394	234 (59.39%)	1.0	XEASY Sparky
CcoNH @ALI	451	315 (69.84%)	563	304 (54.0%)	1.0	XEASY Sparky
HCCHTOCSY @ALI	3443	1004 (29.16%)	2660	737 (27.71%)	1.4	XEASY Sparky
N15HSQC	164	151 (92.07%)	175	139 (79.43%)	1.1	XEASY Sparky
N15NOESY	1774	1177 (66.35%)	4315	999 (23.15%)	1.2	XEASY Sparky
ALL	12519	6842 (54.65%)	18572	5619 (30.26%)	1.2	

Figure S2. Example output of the ARTINA chemical shift assignment application in NMRtist. In addition to the output presented in Figure S1, the application reports a qualitative and quantitative evaluation of the automated chemical shift assignment results. **A**. The diagram presents the protein sequence (140 amino acids) and atoms (rectangles) that can be assigned in the set of NMR spectra given as the application input (panel B, column 'Spectrum name'). Atoms marked in light and dark blue were assigned by the FLYA algorithm with low and high confidence, respectively. **B**. Table with statistical information about number of peaks assigned by FLYA in each of the input spectra. The column 'Expected' presents the theoretical number of cross-peaks that are expected to be present in the spectrum (for NOESY experiments only short-range peaks are included). The column 'Measured' reports the actual number of cross-peaks used for assignment in each spectrum. The columns 'Assigned expected' and 'Assigned measured' report the percentages of assigned 'Expected' and 'Measured' peaks in each spectrum. Overall, the most important quantity is 'Assigned expected' (red box), as it directly reports the amount of information about the system extracted from each spectrum. Since the FLYA algorithm is more robust to false-positives than false-negatives, the number of measured cross-peaks is typically higher than the expected ones. It boosts the overall performance of the method, but results in percentages of 'Assigned measured' peaks to be lower than for 'Assigned expected' peaks.

A

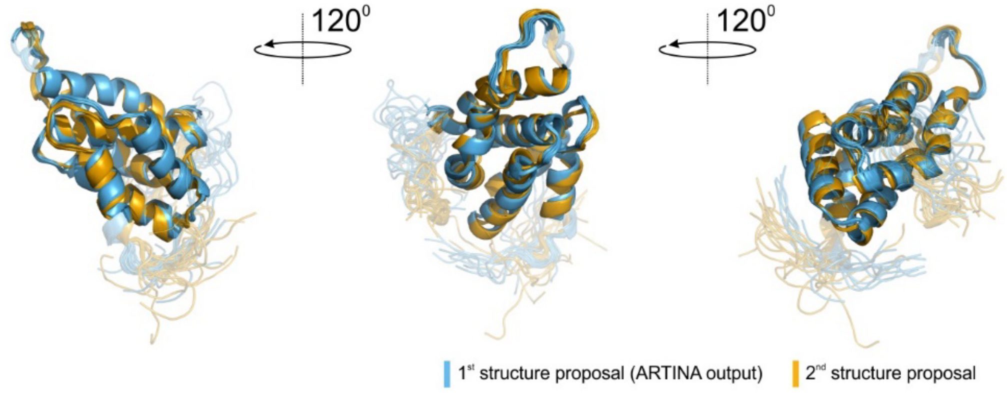

B

Property	1st proposal (ARTINA output)	2nd proposal
Structure	structure.pdb	structure.pdb
Distance restraints	restraints.upl	restraints.upl
Chemical shifts	shift_list.prot	shift_list.prot
Assigned NOESY peaks	Available in results.zip	Available in results.zip
Structure calculation details	cyana_table.txt	cyana_table.txt
Total number of distance restraints	3517	3262
Number of intraresidual restraints ($\|i-j\|$ = 0)	857	836
Number of sequential restraints ($\|i-j\|$ = 1)	900	816
Number of medium-range restraints (1 < $\|i-j\|$ < 5)	932	848
Number of long-range restraints ($\|i-j\|$ ≥ 5)	828	762
Number of torsion angle restraints	212	212
CYANA target function value	7.44 ± 0.26 Å^2	8.76 ± 0.15 Å^2
Distance restraint violations > 0.2 Å	29 ± 2	42 ± 5
Maximal distance restraint violation	0.92 ± 0.00 Å	0.78 ± 0.02 Å
Angle restraint violations > 5.0°	1 ± 1	1 ± 1
Maximal angle restraint violation	5.62 ± 1.03 °	6.08 ± 1.96 °
Residues in most favored Ramachandran plot regions	83.4 %	83.7 %
Residues in additionally allowed regions	15.2 %	15.5 %
Residues in generously allowed regions	1.4 %	0.8 %
Residues in disallowed regions	0.0 %	0.0 %

Figure S3. Example output of the ARTINA automated protein structure determination application in NMRtist. In addition to the output presented in Figures S1 and S2, the application generates visualizations of the protein structure and statistical data about the structure calculation process. **A**. Superposition of the ARTINA output structures (blue, top-ranked structure among 10 candidates calculated internally by ARTINA; orange, second best ARTINA structure candidate). Structures are presented in three different orientations. Regions of the structures shown in intense colours are well-structured. Not well-structured parts of the protein are semi-transparent. By analysing possible discrepancies between the two structure candidates, one can recognise fragments of the protein fold that could not be determined by ARTINA with high confidence. **B**. Table with statistics of the protein structure determination process, which are typically included in a manuscript describing a protein structure determination by NMR.
